# In-hospital mortality and length of stay among patients with infective endocarditis and solid organ transplant: A study from National Inpatient Sample 2016–2019

**DOI:** 10.1016/j.heliyon.2022.e09655

**Published:** 2022-06-04

**Authors:** Nischit Baral, Annabelle Santos Volgman, Tripti Gupta, Arvind Kunadi, Mahin R. Khan, Soumya Kambalapalli, Hameem U. Changezi, Melissa Tracy

**Affiliations:** a Department of Internal Medicine, McLaren Flint/Michigan State University College of Human Medicine, Flint, Michigan, USA; b Department of Internal Medicine, Division of Cardiology, Rush University Medical Center, Chicago, IL, USA; c Department of Internal Medicine, Division of Cardiology, Ochsner Medical Center, New Orleans, LA, USA

**Keywords:** Infective endocarditis, Solid organ transplant, Heart or lung transplant, In-hospital mortality

## Abstract

Infective endocarditis (IE) is a rare but serious complication following a Solid Organ Transplant (SOT). Due to the lack of sufficient studies, we aimed to compare in-hospital mortality and length of stay (LOS) of patients primarily admitted for IE (index or principal hospitalization) with history of SOT, including the subgroup of heart or lung transplant (HLT), to those without a history of SOT (non-SOT) or HLT (non-HLT). We used the 2016–2019 National Inpatient Sample, the largest all-payer inpatient hospital data from Healthcare Cost and Utilization Project (HCUP), including patients 18 years or older with IE, as a principal diagnosis for hospitalization. From 2016 to 2019, there were 56,330 principal or index hospitalizations for IE. Among them, 0.6 % (n = 327) were SOT recipients, 0.1% (n = 68) were HLT recipients, and 41.4% were females. The mean age was 51.9 ± 19.2 years. Compared to non-SOT controls, SOT recipients were older (mean age 59.3 vs. 51.8 years; P = 0.002) and had higher Charlson-comorbidity-index (CCI) of 3 or more (87.7% vs. 33.2%; p < 0.001). SOT status was not statistically significant for a higher or lower odds of in-hospital mortality (adjusted odds ratio (aOR) 0.7; 95% confidence interval (CI): 0.2, 2.4; p = 0.60) or increased or decreased LOS (coefficient: -0.1, 95% CI: -0.4, 0.1; p = 0.23) among index IE hospitalizations after controlling for age, sex, race, hospital-region, hospital-teaching status, income, insurance status, and CCI. HLT status was also not associated with higher or lower odds of in-hospital mortality (aOR 1.4; 95% CI: 0.2, 13.1; p = 0.77) or increased or decreased LOS (coefficient: -0.1, 95% CI: -0.3, 0.5; p = 0.59). From 2016 to 2019, the rate of index IE hospitalization trends from 37.8 to 41.4 per 100,000 overall hospitalizations (p = 0.001). We found the rate of index IE hospitalizations increasing with time. Among index IE hospitalizations, SOT, including a subgroup of HLT recipients, have similar in-hospital mortality and LOS compared to non-SOT or non-HLT groups. We need a larger sample size to comment on outcomes of IE hospitalizations with the HLT subgroup.

## 1 Introduction

Solid-organ transplant (SOT) recipients have complex medical conditions. Due to their frequent contact with health care settings and immunocompromised status, they may be more prone to bloodstream infections [1]. Infective endocarditis (IE) is a rare but severe complication following a SOT. The incidence and prevalence of IE in SOT are unknown since only a few small studies have reported the prevalence of IE in adults with SOT [2, 3]. There is a paucity of data on clinical outcomes (e.g., mortality, length of stay) in this patient population in the United States [4, 5]. Our study aims to bridge the gap, highlighting the association between SOT and outcomes (i.e., in-hospital mortality and length of stay) among patients primarily hospitalized for IE, comparing it to non-SOT IE patients. Since heart or lung transplant (HLT) recipients are a distinct subgroup among SOT patients who carry a very high risk for infections, a sub-group analysis was performed to highlight the outcomes of IE in this population [6].

## 2 Methods

This is a retrospective cohort study of IE hospitalizations using the 2016–2019 National Inpatient Sample (NIS). Since it is delimited de-identified data, the Institution Review Board (IRB) determination from McLaren Health Care (MHC) did not qualify it as human subject research. It exempted the study from oversight by the MHC IRB.

The NIS is part of a family of databases and software tools developed for the Healthcare Cost and Utilization Project (HCUP), Agency for Healthcare Research and Quality (AHRQ) [7]. We collected the data by purchasing and downloading it from the official website of HCUP (www.hcup-us.ahrq.gov). It is the largest publicly available all-payer inpatient healthcare database in the United States. In NIS, the unweighted sample is the discharges that are not yielded to national estimates. In contrast, the weighted model is all the discharges delivered to national estimates and represents all inpatient stays at all the participating hospitals from 50 states of the USA. The unweighted sample contains data from more than 7 million hospital visits each year, and after weighting, it estimates more than 35 million hospitalizations nationally [7]. We analyzed both the unweighted (more minor) NIS data and weighted (more extensive) NIS data.

Our study sample consisted of adults (≥18 years) who were discharged from the hospital with IE as their principal diagnosis and with information on SOT. The index of principal hospitalization is the unique hospitalization for which the patient was primarily admitted or treated for that admission in the hospital (NIS first or central diagnosis variable “I10_DX1”). The diagnostics codes for IE and SOT were based on the International Classification of Diseases, Tenth Revision, and Clinical Modification (ICD-10-CM) diagnoses (Table 1 and Table 2) validated in previously published studies. The final regression models excluded hospitalizations with missing information on our outcomes (in-hospital mortality or LOS) or variables of interest (age, sex, race, CCI, income, hospital region, teaching status, and insurance). For a sub-group analysis, our study sample consisted of adults (≥18 years) discharged with IE as a primary diagnosis and who had undergone either heart transplant or lung transplant or both heart and lung transplant, which were captured using the ICD-10-CM diagnoses codes shown in Table 1 and Table 2.

**Table 1:** ICD-10-CM diagnosis codes and frequencies for various types of Infective Endocarditis.

ICD-10 code	Type of infective endocarditis	Frequency
A32.82	Listerial endocarditis	2 (0.02%)
A39.51	Meningococcal endocarditis	0 (0.00%)
A52.03	Syphilitic endocarditis	2 (0.02%)
A54.83	Gonococcal heart infection	1 (0.01%)
B37.6	Candida endocarditis	71 (0.63%)
I01.1	Acute rheumatic endocarditis	120 (1.07%)
I33.0	Acute and subacute infective endocarditis (Bacterial cause)	9908 (87.95%)
I33.9	Acute and subacute endocarditis, unspecified (Bacterial cause)	422 (3.75%)
I38	Endocarditis, valve unspecified	688 (6.11%)
I39	Endocarditis and heart valve disorders in diseases classified elsewhere	2 (0.02%)
M32.11	Endocarditis in systemic lupus erythematosus	48 (0.43%)

Abbreviations: ICD-10-CM: The International Classification of Diseases, Tenth Revisions, Clinical Modification.

**Table 2:** ICD-10-CM diagnosis codes and frequencies for various types of solid organ transplant (SOT).

ICD-10 Code	Type of SOT	Frequency of SOT among patients with index IE (percent)
Z94.1	Heart transplant	7 (0.06%)
Z94.2	Lung transplant	6 (0.05%)
Z94.4	Liver transplant	9 (0.08%)
Z94.82	Intestine transplant	1 (0.01%)
Z94.0	Kidney transplant	45 (0.40%)
Z94.83	Pancreas	5 (0.04%)
Z94.1 and Z94.2	Heart and lung transplant	13 (0.12%)

Abbreviations: ICD-10-CM: The International Classification of Diseases, Tenth Revisions, Clinical Modification, SOT: Solid Organ Transplant, IE: Infective Endocarditis.

Our outcomes of interest were overall in-hospital mortality and length of stay (LOS) (days). NIS includes a dichotomous variable that identifies if the patient died at the hospital or not (NIS variable DIED); and a continuous variable that represents hospitalization LOS (NIS variable LOS). The in-hospital mortality rate was calculated as the total number of IE patients who died during hospitalization over the total number of IE patients hospitalized during the study period.

The suspected determinant or covariate of central interest was the status of a SOT, including heart, lung, liver, intestine, kidney, or pancreas. A patient was identified with a SOT if they had a history of one or more SOT as a secondary diagnosis during that hospitalization. A patient was placed with an HLT subgroup if they had a history of one or more heart transplants or lung transplants, or both heart and lung transplants. ICD-10-CM diagnosis codes were used to identify SOT (refer to Table 1 and Table 2).

We evaluated the data for outliers and tested the distribution of the LOS. To determine the rate of principal/index IE hospitalization among the study population, we divided the total IE as a principal diagnosis over the total number of discharges in the study period. Similarly, the prevalence of SOT was determined as the total number of patients with at least one SOT over the total number of discharges. To describe the characteristics of the unweighted study sample, our statistical approach consisted of means, medians, interquartile ranges, frequencies, and percentages.

To examine possible group differences (with or without SOT), we used Chi-squared or Fisher's exact with categorical variables (e.g., sex, mortality, race, comorbidity index categories); and t-test and median-test for LOS.

Multivariable logistic regression analysis was performed for the primary outcome of in-hospital mortality. In subsequent research, the statistical approach involved unadjusted and adjusted regression in examining if SOT status was associated with our primary outcomes (i.e., mortality and LOS). We ran negative binomial regression models for LOS as continuous outcomes since our outcomes were nonnegative count variables and had an overdispersion distribution. We defined survey parameters (weight, strata, and stratum) to account for the NIS complex survey sampling methods. For the subgroup analyses, we performed weighted multivariate regression (logistic and negative binomial regressions) in the subgroup of HLT and compared it with non-HLT groups.

We performed investigator-guided analyses to determine the more parsimonious model. The variables included in the initial regression model were socio-demographic characteristics that have been associated with in-hospital mortality or LOS (e.g., age, sex, race, and household income national quartiles) and variables with a p-value of .20 or lower in the unadjusted weighted regressions using forward selection statistical regression methods. We calculated the adjusted odds ratio, which is the odds ratio calculated after adjusting for all the known confounders, including age, sex, race, insurance, hospital location, teaching status of the hospital, income level, year of discharge, and Charlson comorbidity index. We incorporated other variables into the model while testing for statistical significance and evaluating collinearity.

In this analysis, we stressed the precision of the study estimates, focusing on 95% confidence intervals, and p-values will be presented to aid interpretation. All analyses were performed with STATA 17.0 (Stata-Corp LP, College Station, Texas).

## 3 Results

**Figure 1:**
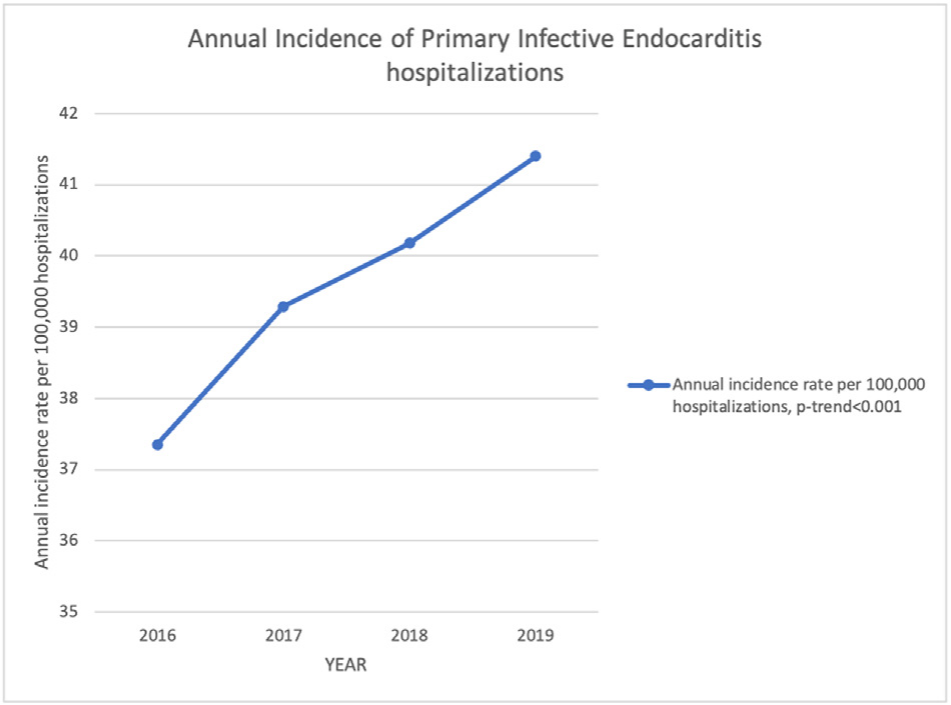
Annual incidence of primary infective endocarditis hospitalization from 2016 to 2019 in the United States.

Our study population consisted of 28,484,087 (unweighted) and 141,900,620 (weighted) hospitalizationsbetween 2016 and 2019. The final study sample consisted of 56,330 (weighted) and 11,266 (unweighted) index hospitalizations with IE as a principal diagnosis. We ended with 39.6 index IE hospitalization per 100,000 hospitalizations. When examining the rate of index IE hospitalization per 100,000 hospitalizations per year (from 2016 to 2019), the rates per year was: 37.8, 39.3, 40.1, and 41.4, respectively (p < 0.001) (Figure 1).

Of the study sample, 88.0% patients had acute or subacute IE (ICD-10 I33.0) which includes bacterial etiology excluding listerial and gonococcal endocarditis; 0.6% had candida endocarditis, 0.02% had syphilitic endocarditis, 0.02% had listerial endocarditis, 0.01% had gonococcal endocarditis, and 1.1% had rheumatic endocarditis. Table 1.

The prevalence of SOT was 0.6% among index hospitalization for IE, of which the most frequent SOT was kidney transplant (0.4%), followed by heart or lung transplant (0.1%), liver transplant (0.1%), pancreas transplant (0.04%), and intestine transplant (0.01%). Table 2.

Table 3 shows the socio-demographic characteristics of our study's unweighted sample (n = 11,266); 41.4% were female, with a mean age of 51.9 years (*SD* = 19.2 years (range 18–90 years). There were 79.1% Whites, 10.9% Blacks, and 8.3% Hispanics. 58.7% lived in areas with first or second median household income in national quartiles; and 37.3% had Medicare, 33.6% Medicaid, and 20.7% had private insurance.

**Table 3:** Characteristic of the unweighted study sample (N = 11266). Adult patients with infective endocarditis as a principal discharge diagnosis, NIS 2016–2019.

	Total N = 11266 % (n)	SOT n = 65 % (n)	Non-SOT n = 11201 % (n)	p-value
*Characteristic*				
Age (mean; SD)	51.88; 19.17	59.31; 11.42	51.84; 19.20	0.002
Sex				
Female	41.38% (4661)	36.92% (24)	41.41% (4637)	
Males	58.62% (6602)	63.08% (41)	58.59% (6561)	0.528
Race				<0.001
White	79.09% (8339)	58.06% (36)	79.21% (8303)	
Black	10.91% (1150)	24.19% (15)	10.83% (1135)	
Hispanic	8.26% (871)	12.90% (8)	8.23% (863)	
Others	1.75% (184)	4.84% (3)	1.73% (181)	
Insurance status				<0.001
Medicare	37.32% (4050)	70.77% (46)	37.12% (4004)	
Medicaid	33.59% (3645)	12.31% (8)	33.72% (3637)	
Private/HMO	20.72% (2248)	16.92% (11)	20.74% (2237)	
Self-pay	8.38% (909)	0.00% (0)	8.43% (909)	
Household income by quartile $				0.455
1-45,999	32.84% (3608)	26.56% (17)	32.88% (3591)	
46k-58,999	25.84% (2839)	28.13% (18)	25.83% (2821)	
59K-78,999	22.90% (2516)	20.31% (13)	22.92% (2503)	
79K or more	18.41% (2023)	25.00% (16)	18.38% (2007)	
Hospital teaching status				0.751
Rural	6.76% (762)	6.15% (4)	6.77% (758)	
Urban (non-teaching)	17.74% (1999)	13.85% (9)	17.77% (1990)	
Urban teaching	75.49% (8505)	80.00% (52)	75.47% (8453)	
Charlson comorbidity index				<0.001
0	27.92% (3146)	1.54% (1)	28.08% (3145)	
1	23.76% (2677)	3.08% (2)	23.88% (2675)	
2	14.8% (1667)	7.69% (5)	14.84% (1662)	
3 or more	33.52% (3776)	87.69% (57)	33.20% (3719)	
Hospital region				0.587
Northeast	22.73% (2561)	16.92% (11)	22.77% (2550)	
Midwest	20.80% (2343)	20.00% (13)	20.80% (2330)	
South	38.32% (4317)	40.00% (26)	38.31% (4291)	
West	18.15% (2045)	23.08% (15)	18.12% (2030)	
In-hospital mortality rate	4.03% (n = 453)	4.62% (3)	4.02% (450)	0.746
Length of stay in days	12.45 ± 13.06	11.63 ± 10.81	12.46 ± 13.08	0.611

Abbreviations: NIS: National Inpatient Sample, SOT: Solid Organ Transplant, HMO: Health Maintenance Organization, SD: Standard Deviation.

We had less than 5% missing data in the bivariate analysis. In-hospital mortality for patients with IE was 4.03% with 4.02% in non-SOT recipients and 4.6% in SOT recipients (p = 0.75). The mean LOS was 12.5 days (SD = 13.1), ranging from 0 to 198 days, with a mean LOS of 12.5 days in non-SOT recipients and 11.6 days in SOT recipients (p = 0.61). The unweighted bivariate analyses suggested no statistically significant group difference (with or without SOT) in sex, income level, hospital teaching status, or region. However, a higher proportion of patients with a SOT were older (mean age 59.3 years vs 51.8 years; p = 0.002), were Blacks (24.2% vs. 10.8%; p < 0.001), had Medicare (70.8% vs. 37.1%; p < 0.001), and had a Charlson-comorbidity-index (CCI) of 3 or higher (87.7% vs. 33.2%; p < 0.001). Table 3.

The bivariate results for the comorbidity suggest that compared to non-SOT, SOT recipients had higher proportion of patients with diabetes mellitus (24.2% vs. 12.7%; p = 0.05), congestive heart failure (46.2% vs. 33.3%; p = 0.03), peripheral vascular disease (15.4% vs. 7.9%, p = 0.04), rheumatoid disease (9.2% vs. 3.2%; p = 0.02), mild liver disease (21.5% vs. 12.1%; p = 0.03), complications of diabetes (35.4% vs. 11.4%; p < 0.001), and chronic kidney disease (86.2% vs. 20.7%; p < 0.001). Table 4. The bivariate analysis results of weighted data were consistent with the unweighted analyses.

**Table 4:** Comorbidities of the unweighted study sample (N = 11266) in adult patients with infective endocarditis as a principal discharge diagnosis, NIS 2016–2019 with and without Solid Organ Transplant (SOT).

Comorbidities in adult patients with IE	Total n (%) in pts with IE	Unweighted frequency in non-SOT n (%)	Unweighted frequency in SOT n (%)	p-value
AMI	958 (8.50%)	950 (8.48%)	8 (12.31%)	0.262
CHF	3760 (33.37%)	3730 (33.30%)	30 (46.15%)	0.034
PVD	898 (7.97%)	888 (7.93%)	10 (15.38%)	0.036
CEVD	1660 (14.73%)	1653 (14.76%)	7 (10.77%)	0.482
Dementia	345 (3.06%)	345 (3.08%)	0 (0.00%)	0.269
COPD	2055 (18.24%)	2046 (18.27%)	9 (13.85%)	0.423
Rheumatoid Disease	367 (3.26%)	361 (3.22%)	6 (9.23%)	0.019
Peptic Ulcer	128 (1.14%)	128 (1.14%)	0 (0.00%)	1.000
Mild liver disease	1369 (12.15%)	1355 (12.10%)	14 (21.54%)	0.033
Diabetes	1194 (10.60)	1178 (10.52%)	16 (24.62%)	0.001
Diabetes with complications	1302 (11.56%)	1279 (11.42%)	23 (35.38%)	<0.001
Cancer	290 (2.57%)	290 (2.59%)	0 (0.00%)	0.417
Hemiplegia/paraplegia	316 (2.80%)	315 (2.81%)	1 (1.54%)	1.00
Renal disease	2379 (21.12%)	2323 (20.74%)	56 (86.15%)	<0.001
Moderate/Sever liver disease	217 (1.93%)	217 (1.94%)	0 (0.00%)	0.639
Metastatic Cancer	122 (1.08%)	122 (1.09%)	0 (0.00%)	1.000
AIDS	90 (0.80%)	90 (0.80%)	0 (0.00%)	1.000

Abbreviations: AMI: Acute Myocardial Infarction, CHF: Congestive Heart Failure, PVD: Peripheral Vascular Disease, CEVD: Cerebrovascular Disease, COPD: Chronic Obstructive Pulmonary Disease, AIDS: Acquired Immuno-Deficiency Syndrome.

There were no differences in the proportion of outcomes and baseline characteristics distribution between the unweighted and weighted samples. The unadjusted univariate regression models suggested that age, race, income level, comorbidity category, and health insurance were associated with in-hospital mortality among IE patients. The final multivariate logistic regression model included SOT status as the main determinant variable with age, race, sex, insurance status, hospital region, hospital teaching status, income level, year, and morbidity category as other covariates. There were 1356 observations (2.4%) missing from the multivariate regression analysis. After controlling for other covariates, SOT status was not significantly associated with in-hospital mortality among IE patients (aOR 0.7; 95% CI: 0.2, 2.4; p = 0.60). However, the results suggested that older IE patients (aOR 1.01; 95% CI: 1.004, 1.02; p = 0.003) have a significantly higher likelihood of in-hospital mortality than younger patients. Similarly, IE patients with CCI of 1 (aOR 2.0; 95% CI: 1.1, 3.6; p = 0.03), CCI of 2 (aOR 2.3; 95% CI: 1.2, 4.3; p = 0.01) or CCI of 3 or higher (aOR 4.5; 95% CI: 2.6, 7.9; p < 0.001) had a statistically significant higher likelihood of in-hospital mortality than patients in the lower morbidity category. Patients in the third (aOR 0.5; 95% CI: 0.4, 0.8; p = 0.01) and fourth (aOR 0.6; 95% CI: 0.4, 0.96; p = 0.03) national quartile of the median household income had a lower likelihood of in-hospital mortality in comparison with the group living in the first quartile. Table 5.

**Table 5:** Adjusted odds ratio of in-hospital mortality among adult patients with infective endocarditis as a principal discharge diagnosis, NIS 2016–2019 (unweighted n = 11266; weighted n = 56330).

In-hospital mortality	Unadjusted Odds Ratios	LL-UL 95% CI	p-value	Adjusted Odds Ratios	LL-UL 95% CI	p-value
Age	1.02	1.02–1.03	<0.001	1.01	1.004–1.02	0.003
Sex (Compared to male(ref)) Female	0.90	0.74–1.08	0.260	0.98	0.80–1.21	0.877
Race (Compared to White (ref))						
Black	1.73	1.31–2.29	<0.001	1.31	0.98–1.76	0.073
Hispanic	1.09	0.75–1.58	0.651	0.89	0.58–1.34	0.567
Other	1.55	0.81–2.95	0.186	1.29	0.64–2.62	0.474
Insurance status (compared to Medicare)						
Medicaid	0.41	0.32–0.52	<0.001	0.85	0.60–1.22	0.381
Private/HMO	0.58	0.44–0.76	<0.001	0.99	0.72–1.37	0.964
Self-pay	0.48	0.32–0.72	<0.001	1.32	0.81–2.16	0.270
Hospital region (compared to Northeast)						
Midwest	1.29	0.95–1.74	0.099	1.22	0.87–1.70	0.244
South	1.03	0.78–1.35	0.849	0.98	0.71–1.34	0.893
West	1.15	0.85–1.56	0.372	1.16	0.82–1.65	0.398
Hospital teaching status (ref. Rural)						
Urban (non-teaching)	0.79	0.47–1.33	0.373	0.84	0.48–1.49	0.553
Urban teaching	1.51	0.97–2.35	0.068	1.55	0.95–2.53	0.082
Household income by quartile $ (compared to (1–45,999)						
46k-58,999	0.94	0.73–1.20	0.613	0.97	0.74–1.27	0.831
59K-78,999	0.70	0.54–0.92	0.011	0.67	0.49–0.90	0.008
79K or more	0.81	0.60–1.08	0.144	0.75	0.53–1.05	0.097
Charlson comorbidity index (compared to 0)						
1	2.98	1.96–4.52	<0.001	2.45	1.53–3.93	<0.001
2	4.47	2.92–6.83	<0.001	3.61	2.24–5.82	<0.001
3 or more	7.85	5.40–11.42	<0.001	5.50	3.52–8.60	<0.001
Year	0.99	0.91–1.08	0.906	1.00	0.91–1.09	0.930
SOT compared to non-SOT	1.15	0.36–3.69	0.808	0.73	0.22–2.42	0.602

Abbreviations: NIS: National Inpatient Sample, SOT: Solid Organ Transplant, HMO: Health Maintenance Organization, SD: Standard Deviation, LL: Lower Limit, UP: Upper Limit, CI: Confidence Interval.

The unadjusted regression results suggested that age, race, hospital region, hospital teaching status, income level, type of health insurance, and morbidity category were associated with differences in LOS among adult patients with IE as the primary diagnosis. There were no significant differences in LOS by SOT status {adjusted coefficient (β) = -0.1; 95% CI: -0.4, 0.01, p = 0.23} among IE patients. The final model suggested that primary IE patients presenting to urban teaching hospital (β = 0.4; 95% CI: 0.3, 0.5, p < 0.001) and urban non-teaching hospital (β = 0.2; 95% CI: 0.1, 0.3, p < 0.001) had longer LOS than rural hospital. Compared to Northeast region, hospitalizations for primary IE in Midwest had shorter LOS (β = -0.1; 95% CI: -0.2, -0.1, p < 0.001) and hospitalizations in South had longer LOS (β = 0.1; 95% CI: 0.01, 0.1, p = 0.02). Moreover, Patients on Medicaid had a longer LOS in comparison with patients covered by Medicare (β = 0.2; 95% CI: 0.1, 0.2, p < 0.001); and the higher the Charlson morbidity category, the longer the LOS among IE patients with CCI of 3 or higher (β = 0.5; 95% CI: 0.5, 0.6, p < 0.001) compared to no comorbidities. Table 6.

**Table 6:** Adjusted and unadjusted coefficient of length-of-stay among adult patients with infective endocarditis as a principal discharge diagnosis, NIS 2016–2019 (unweighted n = 11266; weighted n = 56330).

Length of stay in days	Unadjusted coefficient	LL, UL 95% CI	p-value	Adjusted coefficient	LL-UL 95% CI	p-value
SOT	-0.07	-0.29, 0.16	0.550	-0.14	-0.38, 0.09	0.228
Age	-0.01	-0.01, 0.00	<0.001	-0.01	-0.01, 0.00	<0.001
Sex (compared to male) Female	0.03	-0.01, 0.07	0.156	0.02	-0.02, 0.06	0.227
Race (Compared to White (ref))						
Black	0.08	0.02, 0.12	0.015	-0.03	-0.10, 0.04	0.382
Hispanic	0.06	-0.01, 0.12	0.110	-0.01	-0.08, 0.06	0.711
Other	-0.01	-0.14, 0.13	0.911	0.04	-0.09, 0.17	0.558
Household income by quartile $ (compared to (1–45,999)						
46k-58,999	-0.03	-0.08, 0.02	0.276	0.00	-0.05, 0.06	0.882
59K-78,999	-0.08	-0.14, -0.03	0.004	-0.02	-0.07, 0.04	0.546
79K or more	-0.15	-0.21, -0.09	<0.001	-0.06	-0.12, 0.01	0.081
Hospital region (compared to Northeast)						
Midwest	-0.09	-0.16, -0.02	0.016	-0.12	-0.18, -0.05	0.001
South	0.10	0.04, 0.16	0.001	0.07	0.01, 0.13	0.023
West	-0.05	-0.12, 0.02	0.166	-0.03	-0.10, 0.03	0.328
Hospital teaching status (ref. Rural)						
Urban (non-teaching)	0.27	0.18, 0.36	<0.001	0.21	0.12, 0.31	<0.001
Urban teaching	0.44	0.36, 0.52	<0.001	0.39	0.31, 0.47	<0.001
Insurance (ref. Medicare)						
Medicaid	0.20	0.15, 0.25	<0.001	0.17	0.10, 0.24	<0.001
Private/HMO	0.02	-0.03, 0.07	0.505	0.01	-0.05, 0.07	0.680
Self-pay	0.28	0.20, 0.36	<0.001	0.25	0.16, 0.34	<0.001
CCI (compared to 0)						
1	0.22	0.17, 0.27	<0.001	0.29	0.23, 0.34	<0.001
2	0.25	0.19, 0.32	<0.001	0.40	0.33, 0.46	<0.001
3 or more	0.30	0.25, 0.36	<0.001	0.51	0.46, 0.57	<0.001
YEAR	-0.01	-0.03, 0.02	0.616	-0.01	-0.03, 0.01	0.397

Abbreviations: NIS: National Inpatient Sample, SOT: Solid Organ Transplant, HMO: Health Maintenance Organization, SD: Standard Deviation, LL: Lower Limit, UP: Upper Limit, CI: Confidence Interval.

We performed a subgroup analysis on patients with Heart or Lung Transplant (HLT), which included either heart transplant or lung transplant or heart and lung transplant, and compared them with those without HLT (non-HLT). Among 56330 primary hospitalizations for IE, 0.12% (n = 68) hospitalizations were in patients with HLT. Among HLT hospitalizations, 69.23% were males, 30.77% were females, p = 0.577, 90.91% were Whites, and 9.09% were Blacks, p = 0.748. Compared to non-HLT controls, HLT recipients were older (mean age 59.46 vs. 51.87 years; p = 0.154) and had Charlson-comorbidity-index (CCI) of 3 or higher (76.92% vs. 33.47%; p = 0.020. Table 7. The results of the bivariate analyses of HLT compared to those without HLT are shown in Table 7. HLT status was not associated with higher odds of in-hospital mortality (aOR 1.4; 95% CI: 0.2, 13.1; p = 0.77) or increased LOS (coefficient: -0.1, 95% CI: -0.3, 0.5; p = 0.6) after controlling for age, sex, race, hospital-region, hospital-teaching status, income, insurance status, and CCI (Table 8 and Table 9).

**Table 7:** Characteristic of the unweighted study sample (N = 11266). Adult patients with infective endocarditis as a principal discharge diagnosis, NIS 2016–2019 with Heart or lung transplant (HLT) and without HLT.

	Total N = 11266 % (n)	HLT n = 13 (0.12%)	Non-HLT n = 11253 (99.88%)	p-value
*Characteristic*				
Age (mean; SD)	51.88; 19.17	59.46; 14.12	51.87; 19.17	0.154
Sex				0.577
Female	41.38% (4661)	30.77% (4)	41.40% (4657)	
Males	58.62% (6602)	69.23% (9)	58.60% (6593)	
Race				0.880
White	79.09% (8339)	90.91% (10)	79.08% (8329)	
Black	10.91% (1150)	9.09% (1)	10.91% (1149)	
Hispanic	8.26% (871)	0.00% (0)	8.27% (871)	
Others	1.75% (184)	0.00% (0)	1.75% (184)	
Charlson comorbidity index				0.020
0	27.92% (3146)	7.69% (1)	27.95% (3145)	
1	23.76% (2677)	7.69% (1)	23.78% (2676)	
2	14.8% (1667)	7.69% (1)	14.80% (1666)	
Three or more	33.52% (3776)	76.92% (10)	33.47% (3766)	
In-hospital mortality rate	4.03% (n = 453)	7.69% (1)	4.02% (452)	0.414
Length of stay in days	12.45 SD: 13.06	13.15 SD: 11.82	12.45 SD: 13.07	0.847

Abbreviations: NIS: National Inpatient Sample, HLT: Heart or Lung Transplant, SD: Standard Deviation.

**Table 8:** Adjusted Odds ratio of In-hospital mortality among adult patients with infective endocarditis as a principal discharge diagnosis, NIS 2016–2019 (unweighted n = 11266; weighted n = 56,330) in the subgroup of Heart-Lung transplant.

In-hospital mortality	Unadjusted Odds Ratios	LL-UL 95% CI	p-value	Adjusted Odds Ratios	LL-UL 95% CI	p-value
HLT compared to non-HLT	1.99	0.25–15.58	0.513	1.41	0.15–13.08	0.765

Abbreviations: NIS: National Inpatient Sample, HLT: Heart or Lung Transplant, SD: Standard Deviation, LL: Lower Limit, UP: Upper Limit, CI: Confidence Interval.

**Table 9:** Adjusted and unadjusted Length-of-stay among adult patients with infective endocarditis as a principal discharge diagnosis, NIS 2016–2017 (unweighted n = 11266; weighted n = 56330) in the subgroup of Heart-Lung Transplant.

Length of stay in days	Unadjusted coefficient	LL, UL 95% CI	p-value	Adjusted coefficient	LL-UL 95% CI	p-value
HLT compared to non-HLT	0.55	-0.41, 0.52	0.819	0.11	-0.29, 0.51	0.586

Abbreviations: NIS: National Inpatient Sample, HLT: Heart or Lung Transplant, SD: Standard Deviation, LL: Lower Limit, UP: Upper Limit, CI: Confidence Interval.

### 3.1 Trend analysis from 2016 to 2019

From 2016-to 2019, the rate of primary IE hospitalization trended from 37.8 in 2016 to 41.40 per 100,000 hospitalizations in 2019 (p = 0.001 for all years). Figure 1. Table 10.

**Table 10:** Adjusted mortality and Length of stay in primary IE hospitalizations comparing heart and lung transplant recipients (HLT) vs. non-HLT.

Year	Adjusted In-hospital mortality in HLT (deaths per 100,000 hospitalizations)	Adjusted In-hospital mortality in non-HLT (deaths per 100,000 hospitalizations)	Total in-hospital mortality	P-value
2016	7303	3545	3548	<0.001
2017	7202	3495	3501	<0.001
2018	6539	3163	3167	<0.001
2019	7401	3595	3600	<0.001
Year	Adjusted length of stay in HLT in days (95% CI)	Adjusted length of stay in non-HLT (95% CI)	Total LOS	P-value
2016	14.3 (8.5; 20.0)	11.4 (10.8; 11.9)	11.4 (10.8, 11.9)	<0.001
2017	14.3 (8.5; 20.0)	11.4 (10.9; 11.9)	11.4 (10.9, 11.9)	<0.001
2018	13.8 (8.3; 19.4)	11.0 (10.5; 11.5)	11.0 (10.5, 11.5)	<0.001
2019	14.0 (8.4; 19.6)	11.1 (10.6; 11.7)	11.1 (10.6, 11.7)	<0.001

Abbreviations: HLT: Heart or Lung Transplant, SD: Standard Deviation, LL: Lower Limit, UP: Upper Limit, CI: Confidence Interval.

**Figure 2:**
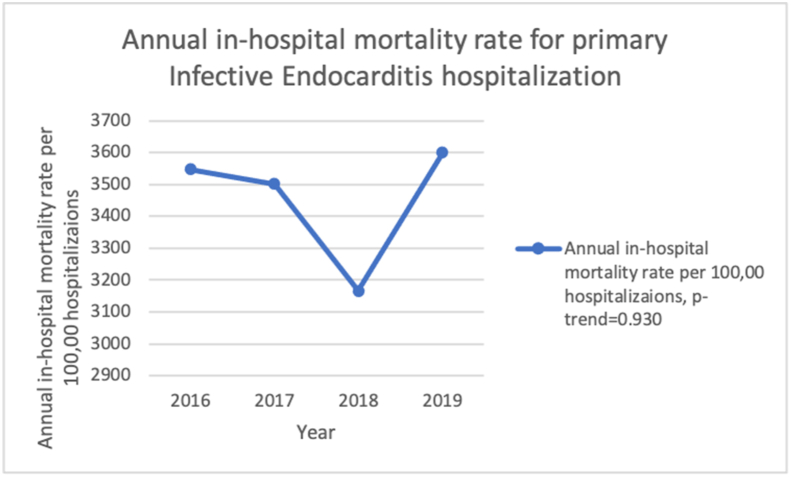
Annual in-hospital mortality rate for primary Infective endocarditis hospitalization from 2016-to 2019 in the United States.

The adjusted in-hospital mortality rate for index IE hospitalizations is 3548 deaths per 100,000 hospitalizations in 2016 to 3600 deaths per 100,000 hospitalizations in 2019 (p-trend = 0.930), with a mean adjusted overall mortality rate of 3454 deaths per 100,000 hospitalizations. Figure 2.

**Figure 3:**
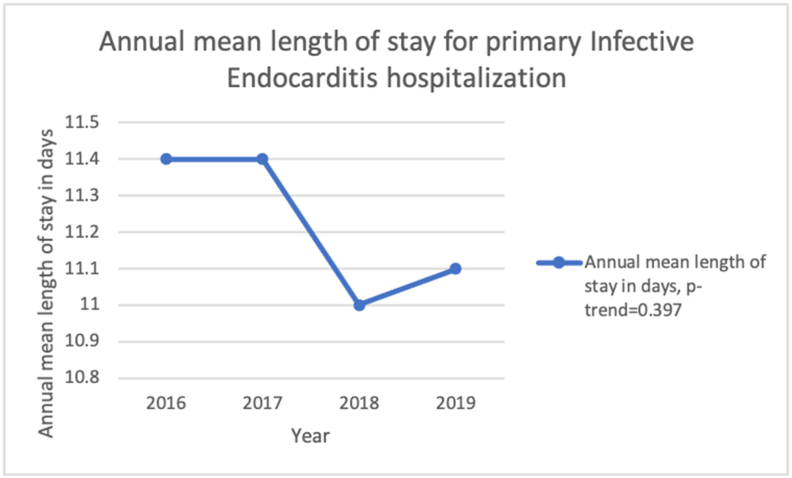
Annual mean length of stay for primary Infective endocarditis hospitalization from 2016 to 2019 in the United States.

The mean LOS decreased from 11.4 days in 2016 to 11.1 days in 2019 (p-trend = 0.397). Figure 3.

**Figure 4:**
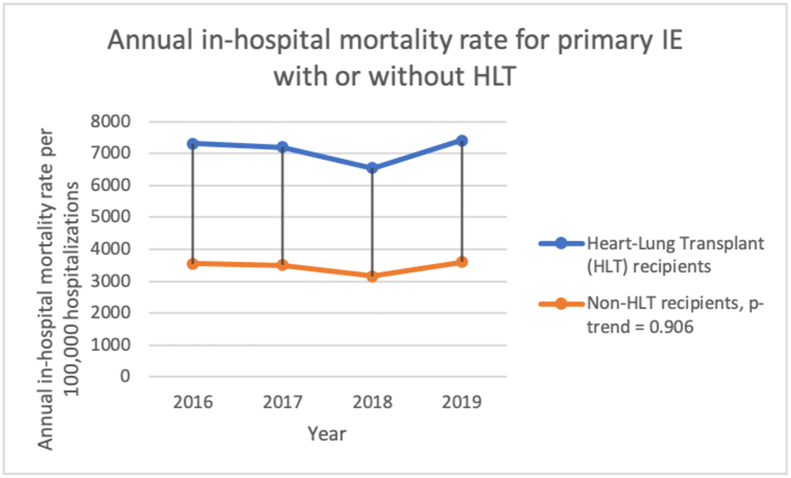
Annual in-hospital mortality rate for primary Infective endocarditis hospitalization for patients with or without heart or lung transplant (HLT) from 2016-to 2019 in the United States.

The trend of adjusted in-hospital mortality among primary IE hospitalization with HLT is 7303 deaths per 100,000 hospitalizations in 2016 to 7401 deaths per 100,000 hospitalizations in 2019, compared to 3545 deaths per 100,000 hospitalizations in 2016–3595 deaths per 100,000 hospitalizations in 2019 in non-HLT, p-values-value-trend = 0.906. Figure 4. Table 10.

**Figure 5:**
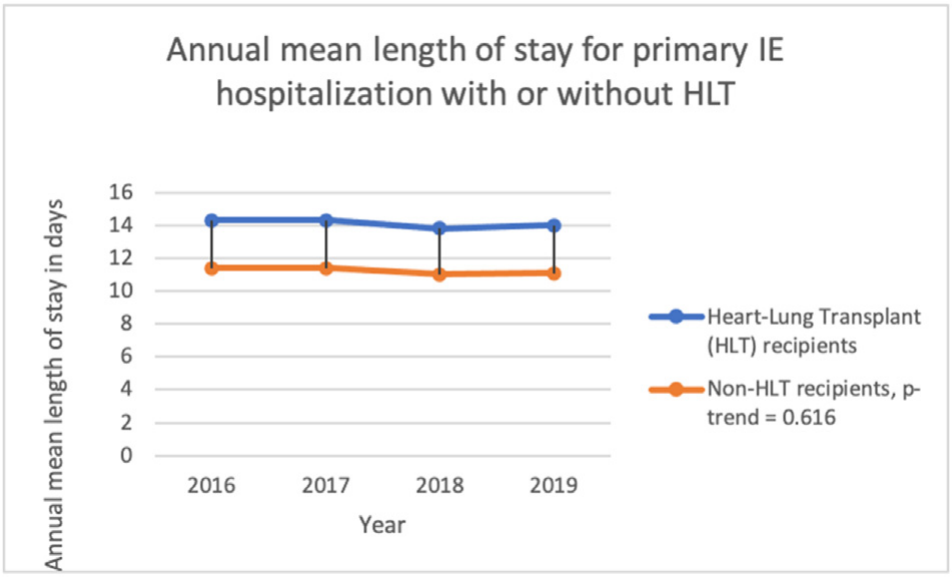
Annual mean length of stay for primary Infective endocarditis hospitalization for patients with or without heart or lung transplant (HLT) from 2016 to 2019 in the United States.

The mean LOS is decreasing from 11.6 days (95% CI: 8.9, 14.3) in 2016 to 11.4 days (95% CI: 8.7, 14.0) in 2019 (p < 0.001) for primary IE hospitalizations with HLT compared to 11.4 days (95% CI: 10.8, 11.9) in 2016 to 11.1 days (95% CI: 10.6, 11.7) in 2019 in non-HLT, p-value-trend = 0.616. Figure 5. Table 10.

**Figure 6:**
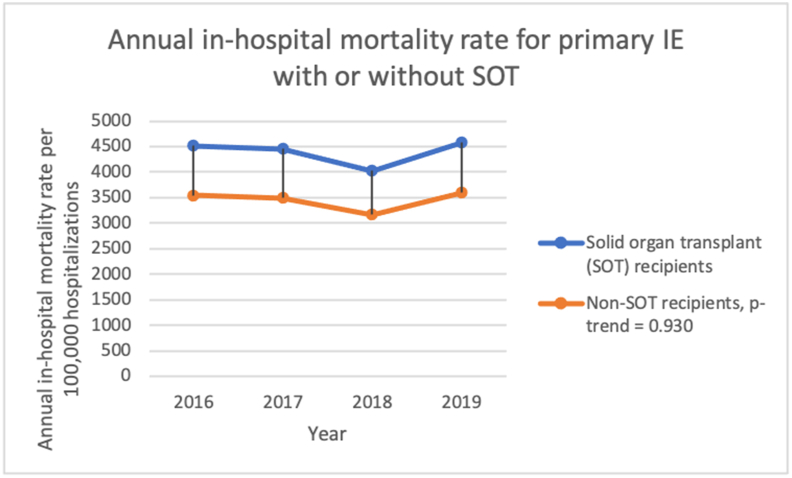
Annual in-hospital mortality rate for primary Infective endocarditis hospitalization for patients with or without solid organ transplant (SOT) from 2016-to 2019 in the United States.

The mean LOS is decreasing from 11.6 days (95% CI: 8.9, 14.3) in 2016 to 11.4 days (95% CI: 8.7, 14.0) in 2019 for primary IE hospitalizations with SOT, compared to 11.4 days (95% CI: 10.8, 11.9) in 2016 to 11.1 days (95% CI: 10.6, 11.7) in 2019 in non-SOT, p-value-trend = 0.397. Figure 6. Table 11.

**Table 11:** Adjusted mortality and Length of stay in primary IE hospitalizations comparing solid organ transplant recipients (SOT) vs. non-SOT.

Year	Adjusted In-hospital mortality in SOT (deaths per 100,000 hospitalizations)	Adjusted In-hospital mortality in non-SOT (deaths per 100,000 hospitalizations)	Total in-hospital mortality	P-value
2016	4513	3543	3548	<0.001
2017	4452	3496	3501	<0.001
2018	4024	3162	3167	<0.001
2019	4580	3595	3600	<0.001
Year	Adjusted length of stay in SOT in days (95% CI)	Adjusted length of stay in non-SOT (95% CI)	Total LOS	P-value
2016	11.6 (8.9, 14.3)	11.4 (10.8, 11.9)	11.4 (10.8, 11.9)	<0.001
2017	11.6 (8.9, 14.3)	11.4 (10.9, 11.9)	11.4 (10.9, 11.9)	<0.001
2018	11.3 (8.6, 13.9)	11.0 (10.5, 11.5)	11.0 (10.5, 11.5)	<0.001
2019	11.4 (8.7, 14.0)	11.1 (10.6, 11.7)	11.1 (10.6, 11.7)	<0.001

Abbreviations: SOT: Solid Organ Transplant, SD: Standard Deviation, LL: Lower Limit, UP: Upper Limit, CI: Confidence Interval.

**Figure 7:**
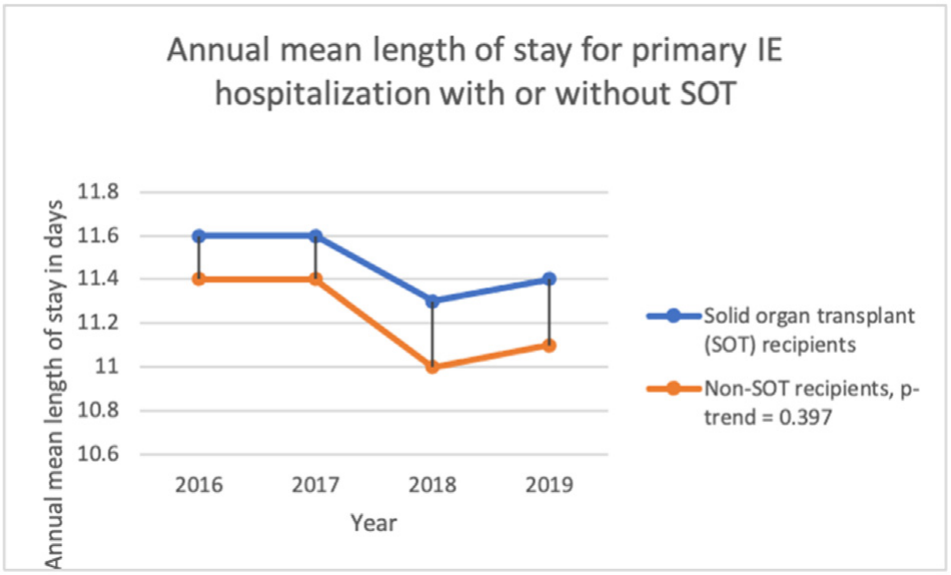
Annual mean length of stay for primary Infective endocarditis hospitalization for patients with or without solid organ transplant (SOT) from 2016-to 2019 in the United States.

The trend of adjusted in-hospital mortality among primary IE hospitalizations with SOT is 4513 deaths per 100,000 hospitalizations in 2016 to 4580 deaths per 100,000 hospitalizations in 2019, compared to 3543 deaths per 100,000 hospitalizations in 2016–3595 deaths per 100,000 hospitalizations 2019 in non-SOT, p-value-trend = 0.930. Figure 7. Table 11.

## 4 Discussion

Our 2016–2019 NIS database results showed a rising principal IE hospitalization rate annually. We further looked into the SOT and HLT as contributing factors.SOT is not common among index IE hospitalizations in the US, with rate of 0.6%. Among index IE hospitalizations with SOT, most patients were older with a higher prevalence of comorbidities such as kidney disease, diabetes mellitus, congestive heart failure, peripheral vascular disease, rheumatoid disease, liver disease, and chronic kidney disease and quantified collectively by CCI. Despite this, they did not have worse healthcare outcomes, such as higher in-hospital mortality or longer LOS. Similarly, the in-hospital mortality rate and LOS were not significantly different in a subgroup analysis of index IE hospitalizations with or without HLT.

Our findings are similar to the study done by Eichenberger et al. [8], a retrospective study of a nationwide readmission database from 2013 to 2018, which reported that the in-hospital mortality rate was similar in SOT and non-SOT groups. In our research, we did not look exclusively into the index solid organ transplantation procedural hospitalizations and only included index hospitalizations with IE. The in-hospital mortality rate of index IE hospitalization in SOT recipients in our study (4.6%) is lower than expected compared to other studies (14%–64%) [3, 4, 5, 8]. This may be due to our study's exclusion of index SOT procedural hospitalization. Moreover, our analysis captures data from 2016 to 2019, which may be reflective of advances in the medical diagnoses and management of SOT recipients, and utilization of specialized transplant clinics, as explained in the annual report from the Organ Procurement Transplant Network (OPTN)/Scientific Registry of Transplant Recipients (SRTR), which reports improved quality of care and survival in the solid organ recipients [9, 10, 11, 12, 13]. Another study in a national sample in Spain by Martínez-Sellés et al. [4] also showed no significant differences in in-hospital mortality between SOT versus without SOT groups.

Our study contrasts with an investigation by Chuang et al., who reported the 30-day mortality to be higher (14%) in the SOT recipient group compared to the non-SOT group (0%). However, the sample size in the study was small (14 cases of IE with SOT) [5]. Ruttman et al. [2] reported a single-center survey with 27 points of IE in SOT recipients. In a two-center study, Paterson et al. [3] reported 14 cases of IE in kidney and liver transplant recipients, where mortality ranged from 44-64%, which is drastically different from our findings of 4.6%. This is probably related to many of their cases coming from autopsies, while our study included hospitalization data in the real world [2, 3]. In the subgroup of HLT, the mortality rate was 7.9% which is comparable to the annual report of OPTN/SRTR, where the one-year mortality in 2018 for heart transplant recipients was 7.9% [9]. Our results also show that in 2018, there was a decrease in the annual in-hospital mortality rate and LOS. In 2018, there was a decrease in the yearly in-hospital mortality rate and LOS, likely explained by the increase in the number of patients getting SOT in 2019 compared to 2018 [10, 11, 12, 13].

While the in-hospital mortality may not be as alarming, we know that patients with SOT are chronically immunosuppressed and at high risk for infections, including bacterial septicemia and pulmonary diseases in the first- and 6-months post-transplant, contributing to the overall cost of healthcare and morbidity. Common organisms that cause IE in SOT recipients include Staphylococcus aureus, Aspergillus fumigatus, Enterococcus species, and Candida species. Still, there are no specific guidelines for the management of IE in SOT patients [14]. Among index IE hospitalization with SOT, 69.7% were kidney transplant recipients. This increased risk may be due to the higher risk of infection from vascular access sites (arterio-venous grafts), hemodialysis in these patients before transplant causing valvular calcification, and increased risk of hospital-acquired disease [15, 16]. Given the high prevalence of IE in SOT patients, our study emphasizes the need to educate patients and providers to maintain a high level of suspicion for IE when they present to the hospital.

Our study is one of the few studies to highlight the outcomes of index IE in hospitalizations of patients with a history of SOT or HLT from the latest NIS database, including trends in in-hospital mortality and LOS. The similar in-hospital mortality and LOS among primary IE hospitalization with SOT compared to the non-SOT group may reflect SOT recipients’ improved quality of care and management [9, 10, 11, 12, 13]. Most other published studies on heart transplants or lung transplants are either case series or case reports [17, 18, 19]. With the most recent available database, our study used a much larger population than analyzed in previously published studies. The in-hospital mortality in the HLT group was much higher than in the non-HLT group, but the result was not statistically significant. The nonsignificant result in our study may be due to the small sample size of just 68 hospitalizations in the HLT group. The rate of in-hospital mortality and LOS are similar from 2016-to 2019, which further suggests that recently there may have been stagnation in the improvement in the quality outcomes in these patient populations, which is further proved by the study by Rana et al., who reports that there is a plateau in survival in SOT recipients in the recent years [20]. Moreover, as our research shows, more than 90% of the index hospitalizations are due to bacterial causes; appropriate use of antibiotics with proper antibiotic stewardship to prevent antibiotics resistance may be vital to improving survival from the current plateau phase of survival in solid organ transplant recipients in the recent years [20].

In the future, studies with larger sample sizes, especially in the HLT group, are needed to address the gap in the literature and update the guidelines on the management of IE in patients with HLT. Individual solid organ transplant pathologies and different organisms involved in the IE can provide valuable information in future studies on the topic. Left and right heart endocarditis should also be studied separately in future studies.

The limitations of this study should be noted. The data were mainly obtained from NIS and might be prone to various biases, such as selection bias, given that not all hospital systems are part of the NIS. There may also be errors associated with documentation and data capture from a database. Our data regarding the rate of IE by SOT type are, for sure, an underestimation. We have not done subgroup analysis on SOT, including heart, lung, heart and lung, liver, kidney, heart and kidney, heart and liver, pancreas, intestinal, etc. That would make the manuscript very lengthy. However, each solid organ transplants have different pathologies with different comorbidities and demographics, thus may have other clinical utility, which is one of the significant limitations of our study. NIS dataset doesn't provide information about the length of time post-transplant, details on immunosuppression, transplant rejection, and therapies utilized, which is another limitation of our study. The definitions used to diagnose IE might be inaccurate as these are the limitation of data from NIS, HCUP, and AHRQ [7].

We found the rate of principal IE hospitalizations increasing with time. Among patients hospitalized for IE, SOT, including a subgroup of HLT recipients, have similar in-hospital mortality and LOS compared to the non-SOT or non-HLT group. We need a larger sample size to comment on outcomes of IE hospitalizations with HLT.

## Declarations

### Author contribution statement

Nischit Baral and Annabelle S Volgman: Conceived and designed the experiments; Performed the experiments; Analyzed and interpreted the data; Contributed reagents, materials, analysis tools or data; Wrote the paper.

Tripti Gupta and Arvind Kunadi: Analyzed and interpreted the data; Contributed reagents, materials, analysis tools or data; Wrote the paper.

Mahin Khan; Soumya Kambalapalli; Hameem Changezi and Melissa Tracy: Analyzed and interpreted the data; Contributed reagents, materials, analysis tools or data; Wrote the paper.

### Funding statement

Volgman was supported by 10.13039/100000002NIH IND [Number 119127; NIH NINR R01NR018443].

### Data availability statement

The authors do not have permission to share data.

### Declaration of interest’s statement

The authors declare no conflict of interest.

### Additional information

No additional information is available for this paper.
